# Quantification and characterization of collagen in experimentally induced wounds treated with different types of platelet-rich plasma gel

**DOI:** 10.1007/s10561-026-10215-5

**Published:** 2026-03-04

**Authors:** G. Haro de Melo, F. M. Moreira, G. G. Mori, L. F. Bento, D. V. Barrionuevo, C. B. Laposy, R. Giuffrida, A. F. F. Rodrigues, G. A. T. Ozaki, D. A. F. da Silva, F. L. Pacagnelli, R. M. B. Nogueira

**Affiliations:** 1 Medicine Course of Presidente Prudente, University of Western São Paulo, St Jose Bongiovani, 700-CEP 19050-920, Presidente Prudente, São Paulo, Brazil; 2 Graduate Program in Animal Sciences, University of Western São Paulo, Presidente Prudente, São Paulo, Brazil; 3 Paulista University, Araçatuba, São Paulo, Brazil; 4 Veterinary Medicine Course of Presidente Prudente, University of Western São Paulo, Presidente Prudente, São Paulo, Brazil

**Keywords:** Fractal dimension, Platelet-rich plasma, Collagen, Healing, Rabbit

## Abstract

Due to its high concentration of growth factors, platelet-rich plasma (PRP) has become the subject of much research, either as an isolated or adjuvant treatment for wound repair. The fractal dimension has been used as an innovative method for the characterization and quantification of tissue structural alterations without any interference from the evaluator. The objective of this study was characterizing collagen types I and III in experimentally induced wounds and treated with different PRP sources by means of fractal dimension and other histological analyzes using blades stained with picrocirius-red and hematoxylin-eosin (HE). In statistical analysis, the variables presented a non-parametric distribution; thus, Mann-Whitney and Kruskall-Wallis tests (*p* < 0,05) were performed using the Student-Newman-Keuls method. It was observed an increase of the collagen fibers by measuring the area with Threshold Color in the different treatments, especially with autologous PRP (*p* = 0.009) and heterologous PRP (*p* = 0.016). The fractal dimension of type I collagen had a significant increase for the autologous group (*p* = 0.028) in relation to the control, but, on average (*p* < 0.05), type III collagen was higher than type I. Fractal dimension was efficient to separately characterize type I and type III collagen fibers. In this study, collagenization was higher in autologous when compared to heterologous and homologous PRP treatments.

**Supplementary Information:**

The online version contains supplementary material available at 10.1007/s10561-026-10215-5.

## Introduction

Platelet-rich plasma (PRP) has recently become the focus of many studies for its roles in tissue repair; it contains a high concentration of platelets, and is rich in growth factors and cytokines, which benefits the process of repair or tissue healing (Vendramin et al. 2010; Fang et al. 2020; Gupta et al. 2021; Gabusi et al. 2024; Sharara et al. 2021). According to Wu et al. (2025), in 2025, a total of 200,000 platelets are present in 1 µl of PRP, representing an increase of two to five times compared with blood, as well as a three- to fivefold increase in the concentration of growth factors, especially Vascular Endothelial Growth Factor and Platelet-Derived Growth Factor.

Growth factors are released by the degranulation of activated platelets and by cells at the lesion site to promote cell migration and proliferation, resulting in neovascularization and the formation of granulation tissues essential for wound repair (Paichitrojjana and Paichitrojjana 2022). Such granules contained within the platelets contain transforming growth factor beta (TGF-β), insulin-like growth factor I (IGF-I), platelet-derived growth factor (PDGF), vascular endothelial growth factor (VEGF), fibroblast growth factor (FGF), epidermal growth factor (EGF) (Wu et al. 2025; Sharun et al. 2021; Xu et al. 2020), and connective tissue growth factor (CTGF) (Maia and Souza 2009), all of which are important for tissue repair (Verma et al. 2023).

PRP gel can be obtained from several sources: autologous PRP is prepared from own blood, homologous PRP is extracted from the blood of another individual of the same species, and heterologous PRP is obtained from blood extracted from different species (Vendramin et al. 2010). And, numerous studies have demonstrated the effects of PRP on healing, reporting increased collagen production in dermal wounds (Abegão et al. 2015; Marques et al. 2017; Zein AlAbidden and Vandeputte 2025), burns (Zheng et al. 2022), muscle lesions (O'Dowd 2022; Vale et al. 2024), periodontal diseases (Xu et al. 2020; Tobita et al. 2026; Gawlak-Socka et al. 2026), bone defects (Yang et al. 2026), endometrial disorders (Karadbhajne et al. 2024), lumbar disc herniation (Guo et al. 2025), among other.

However, there are few studies evaluate the quality of scar tissue and of the collagen in wounds treated with different types of PRP; collagen is an important protein that plays an essential role in the elasticity and resistance of the skin, is abundant in tissues during healing, and is a primary component of the extracellular matrix (ECM) (Pu et al. 2023; Diller and Tabor 2022). In the tegument, collagen fibers represent 50–90% of your composition; type I collagen, the most predominant type, constitutes 80–85% of dermal collagen, while type III makes up approximately 8–11% (Gardeazabal and Izeta 2024).

An innovative method that allows evaluation without interference by the evaluator, called the dimensional fraction (DF), has been used on histological slides, and can be used for the characterization of irregular and complex structures, as well as the quantification of alterations (Lima et al. 2023; Weber et al. 2024; Tambasco et al. 2009; Meregalli et al. 2022). With polarization microscopy, the Picrosirius-stained collagen fibers formed a dense network characterized by the birefringent properties of the collagen molecule (Lattouf et al. 2014), enabling the differentiation between immature and mature collagen. In the red, green, blue (RGB) system, mature collagen fibers (type I) appear strongly birefringent and thicker, juxtaposed with yellow, orange, and red color variations, while immature fibers (type III collagen) are thinner and more sparse, weakly birefringent, and greenish in color (Santinoni et al. 2020).

For DF analysis, the images were passed through the Split Channel process, which divides the channels of the image. RGB image colors were used to display multi- channel images. The colors were designed to reflect the true colors of the samples. Using the Split Channel function, an RGB image can be divided into its respective monochromatic components: red, green, and blue (Color Image Processing 2026). Later, these images were binarized to black and white; this procedure was necessary because DF analysis measures the black area of the image (Abonizio et al. 2022).

This method was adopted because it allowed us to quantify the area occupied by each type of collagen fiber in the histological section. Generally, a greater fractal dimension indicates that the shape has a greater complexity and requires more complex calculations; the box- counting technique is the most useful for this type of analysis (Jeong et al. 2023).

We hypothesized that DF may be useful as an auxiliary method to evaluate the pathophysiology of tissue repair, as it can be an important factor in determining the most appropriate therapeutic protocol for wound healing using different sources of PRP.

The objective of this study was quantified and characterized the collagen in the tissue repair of experimentally induced wounds treated with different sources of PRP gel through histological and fractal analysis.

## Materials and methods

### Animal models

Prior to the experiment, held sample design in order to minimize the number of animals used. This study has been approved by the Ethics Committee (Protocol n°. 3520) and was conducted in accordance with ethical principles.

Twenty-four healthy New Zealand rabbits (twelve males and twelve females), aged 150 days old, with an average weight of 3.0 ± 1.0 kg, were used; where six rabbits (three males and three females) were used as donors for the homologous PRP gels. A healthy, unprocessed dog, aged 6 years old and weighing 25 kg, was used as a donor for the heterologous PRP gel. The animals were maintained in a controlled temperature of 25 ± 2 °C, a relative humidity of 50 ± 15%, and a normal photoperiod (12–12 h light–dark cycle). All animals received solid feed, except during the first twelve preoperative hours, and water *ad libitum.*

### Preparation of PRP

After the rabbits were anesthetized intramuscularly with Tiletamine and Zolazepan (Zoletil 50, Virbac, São Paulo, Brazil) at a dose of 15 mg/kg, 4 mL blood was collected from the atrial vein for preparation of the autologous and homologous PRP gels. The same volume of blood was collected from the jugular vein of the dog, using a vacuum collection tube with EDTA as an anticoagulant, to prepare the heterologous PRP gel. Blood samples were collected through automatic plate counting (Automatic Sysmex Poch Diff 100i, V-Roche Diagnostica, São Paulo, Brazil) to obtain the initial whole- blood platelet values. Blood samples were centrifuged at 200× *G* (Excelsa Baby Centrifuge 206R, São Paulo, Brazil) for 10 min, forming a lower layer containing red blood cells, an intermediate layer called the mist zone, and an upper layer containing plasma and platelets. The plasma layer, along with 200 μL of the mist zone, was transferred to a clean tube and centrifuged at 400× *G* for 10 min, forming platelet-poor plasma (PPP) in the upper layer and the erythrocytic-platelet bud in the lower layer. PPP was discarded, and platelet counts were performed through both manual and automatic methods using a hemocytometer (Boeco, Hamburg, Germany) to ensure that the platelet concentration was at least six-fold higher than in blood (Zheng et al. 2022). Once the appropriate concentration was obtained, the liquid PRP was transformed into a gel through the addition of 10% calcium gluconate at a ratio of 4:1 (Margono et al. 2022). The final volume of all the PRP gels was 0.5 mL (Zheng et al. 2022).

### Induction and treatment of wounds

The animals were submitted to trichotomy in the dorsal region near the insertion of the scapula with an electric trichotomizer (Andis AGC2-USA) and slide no. 40. They were then anesthetized intramuscularly with tiletamine and zolazepam (Zoletil 50, Virbac, Brazil) at a dose of 15 mg/kg (Cinar et al. 2024). Skin antisepsis with 70% ethanol was performed, followed by the administration of 1.0 mL of local anesthetic (2% lidocaine with a vasoconstrictor) into the subcutaneous tissue. With the aid of a sterile 8-mm-diameter punch, a fragment of skin was removed and the subcutaneous tissue was dissected using blunt-ended scissors, preserving the left and right musculature.

**Fig. 1:**
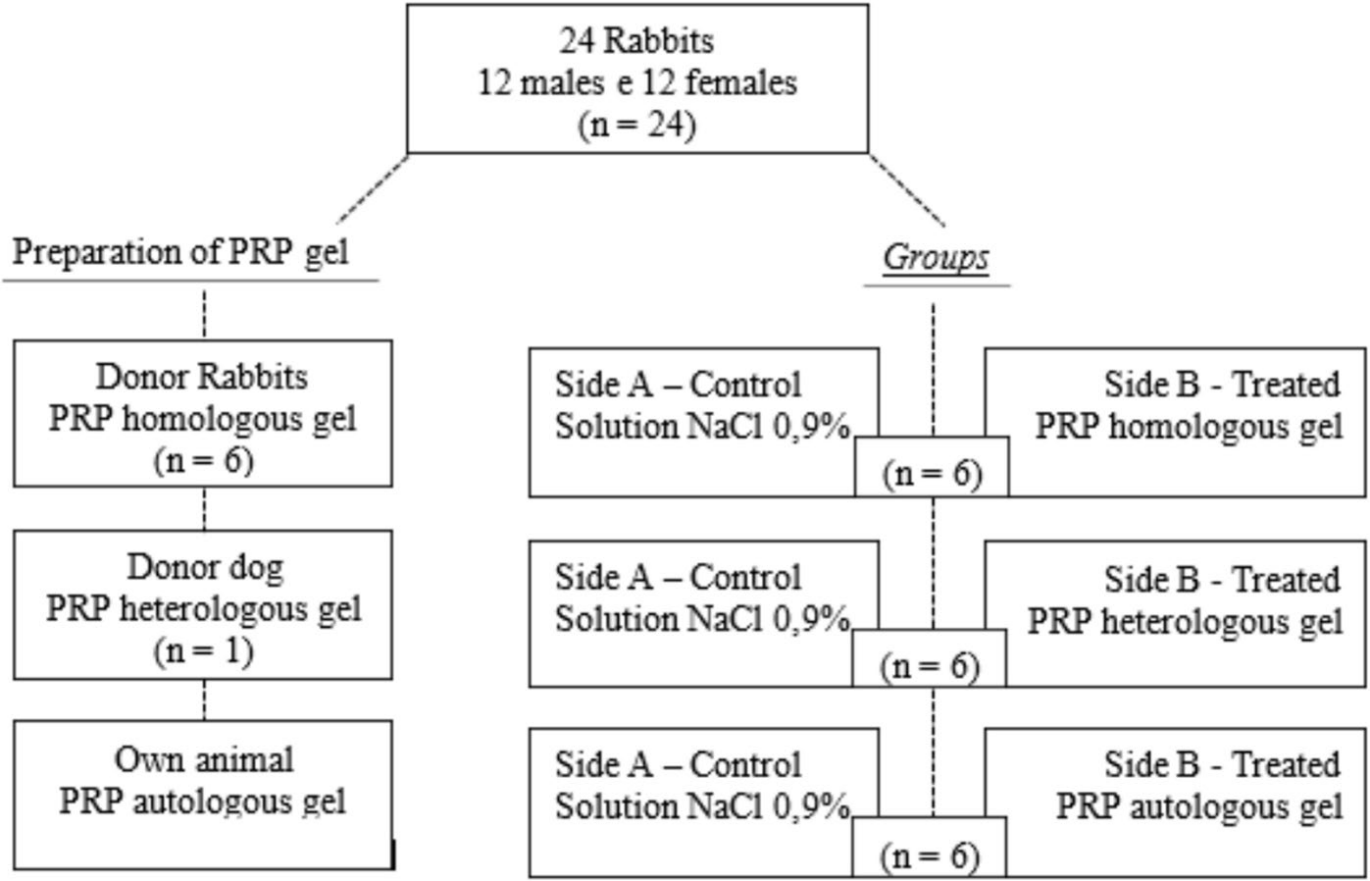
Representation of the distribution of animals by procedure and division of groups

The left side (A) was treated with 0.9% saline solution (Fresenius, Germany) in all animals and was considered the control group (Solution NaCl 0.9%) for the therapies analyzed in this study. The wounds on the right side (B) were treated with PRP gel and divided into three experimental groups, designated as PRP autologous gel group (n = 6), PRP homologous gel group (n = 6), and PRP heterologous gel group (n = 6), as can be observed in Figure 1. For this purpose, rabbits were allocated randomly according to the origin of the PRP applied to the wounds.

Thus, the wounds were treated with 0.9% saline solution or PRP gel, as previously described, on days 0, 3, 7, 10, and 14. Prior to the topical application of saline solution or PRP gel, the wounds were cleaned with the aid of sterile gauze soaked in 0.9% saline solution. For the application of saline solution in the control group, a sterile disposable syringe was used, and PRP gel was applied with the aid of a straight Kelly forceps (Golgran, São Caetano do Sul, SP). After each treatment, lesions were protected with sterile rayon (1.0 cm^2^) and hypoallergenic tape immediately after treatment; this was replaced at regular intervals with a circular adhesive.

Additionally, the animals received tramadol (Teuto Brasileiro S/A, Goiás, Brazil) at a dose of 0.5 mg/kg intramuscularly every 12 h for 3 consecutive days after wound creation for pain control.

### Histological processing and evaluation of the samples

On day 17 (the final day of the experiment), after sedating the animals using the same wound induction protocol described above, a biopsy was performed by extracting the tissues ranging from the central area to the wound edges using an 8-mm diameter punch. The excised skin fragments were fixed in 10% formalin solution for 24 h and subsequently embedded in paraffin. Section 5 μm thick were obtained and stained with Hematoxylin and Eosin (H&E) and Picrosirius red F3BA, a specific and efficient staining method used to demonstrate the organization and heterogeneity of collagen fibers (López De Padilla et al. 2021).

### Fractal dimension analysis

The Picrosirius-stained slides were photographed using a LEICA (DFC 450) polarized light microscope and digital images were analyzed using the Leica Application Suite. All images were obtained at 40×  magnification. For the fractal dimension, 6 histological fields of the lesion area of each animal were photographed. All procedures for DF analysis were performed using Image J software (http://rsbweb.nih.gov/ij/). Because of the Picrosirius staining, the collagen fibers presented with predominantly red and green coloration.

Finally, DF analysis was performed using the box-counting method. The program considers two dimensions, allowing the quantification of pixel distribution in the space, and does not consider the texture of the image (Abonizio et al. 2022). This allows two images with the same distribution of pixels, one binarized and the other in grayscale, to have the same DF. Thus, the DF calculated by ImageJ will always be between 0 and 2, and does not distinguish different textures (Nai et al. 2021).

### Collagen area

In order to measure the collagen area, the images were analyzed using the Image J Threshold Color plugin; through automated particle selection and measurement of the color-based area (Sharun et al. 2023), the collagen area was obtained. The values for collagen fibers in the Threshold Color plugin were standardized for all the images.

### Fibroblast counts

The blades stained with HE was photographed with a Leica DM750 microscope and digital images were analyzed with the Leica Application Suite image analysis program. The scanned microscopic images were obtained at 40×  magnification.

Fibroblast counting was performed under an optical microscope; fibroblasts are an important cell type involved in the synthesis of collagen, supporting the extracellular matrix (Boraldi et al. 2024). Cell counting was performed manually with the ImageJ Cell Counter plugin, which was used to mark the objects of interest in the image. We analyzed 10 histological fields of the lesion area of each animal and later calculated the group average. Also, in these slides, areas of necrosis or inflammation were analyzed.

### Statistical analysis

All data groups were submitted to the Shapiro–Wilk test to validate the data normality assumption, whereby the majority of the variables presented a non-parametric distribution. Thus, control and treated groups were compared using the Mann-Whitney test, different treatments were compared using the Kruskall-Wallis test, and the Student-Newman-Keuls method was used to compare results within each group. The relationship between the different variables was evaluated by Spearman’s non-parametric correlation analysis. The analyses were conducted using software R version 33.0 and additional packages (R DEVELOPMENT CORE TEAM 2026). A value of *p* < 0.05 was considered statistically significant.

## Results

In the microscopic analysis, the coloration of Picrosirius under polarized light marked type I collagen with orange and red birefringence, and marked type III collagen with green birefringence. In the treated groups, type I collagen fibers were more abundant and organized than type III fibers, which were more irregularly distributed.

According DF analysis showed that the median of collagen type III was higher than type I (Table 1) in all three treated groups, being higher in the heterologous group (*p* < 0.05). In addition, a Fractal analysis of type I collagen demonstrated a significant statistical difference (*p* = 0.028) compared to the control, but only within the autologous group.

**Table 1:** Fractal dimension, collagen area and fibroblast counts of the autologous, homologous and heterologous groups. Comparison of the medians (minimum–maximum values) between control (A) and treated (B) groups

	A		B		
	Medians	Min–Max	Medians	Min–Max	*p* (value)
*Autologous*					
DF type I	1,41	(1,40–1,44)	1,45	(1,41–1,53)	0,028
DF type III	1,78	(1,75–1,85)	1,76	(1,70–1,90)	0,347
Area (pixel)	15,923	(11,101–17863)	24,408	(18,638–294,370	0,009
Fibroblasts	86,80	(46,10–94,90)	63	(30,50–86,50)	0,174
*Homologous*					
DF type I	1,43	(1,36–1,53)	1,46	(1,28–1,49)	0,916
DF type III	1,69	(1,58–1,85)	1,74	(1,60–1,81)	0,916
Area (pixel)	17,569	(14,429–28,460)	16,760	(13,274–19,765)	0,347
Fibroblasts	53,10	(27,30–86,50)	36,70	(24,30–54,90)	0,250
*Heterologous*					
DF type I	1,41	(1,40–1,53)	1,43	(1,37–1,46)	0,676
DF type III	1,71	(1,61–1,85)	1,78	(0,91–1,86)	0,916
Area (pixel)	8355	(20,302–8116)	21,034	(17,821–26,849)	0,016
Fibroblasts	86,70	(40,30–96,50)	83,90	(46–118,80)	0,916

DF type I = fractal dimension collagen type I; DF type III = fractal dimension collagen type III; Area = total area of collagen per group. *p* < 0,05

**Fig. 2:**
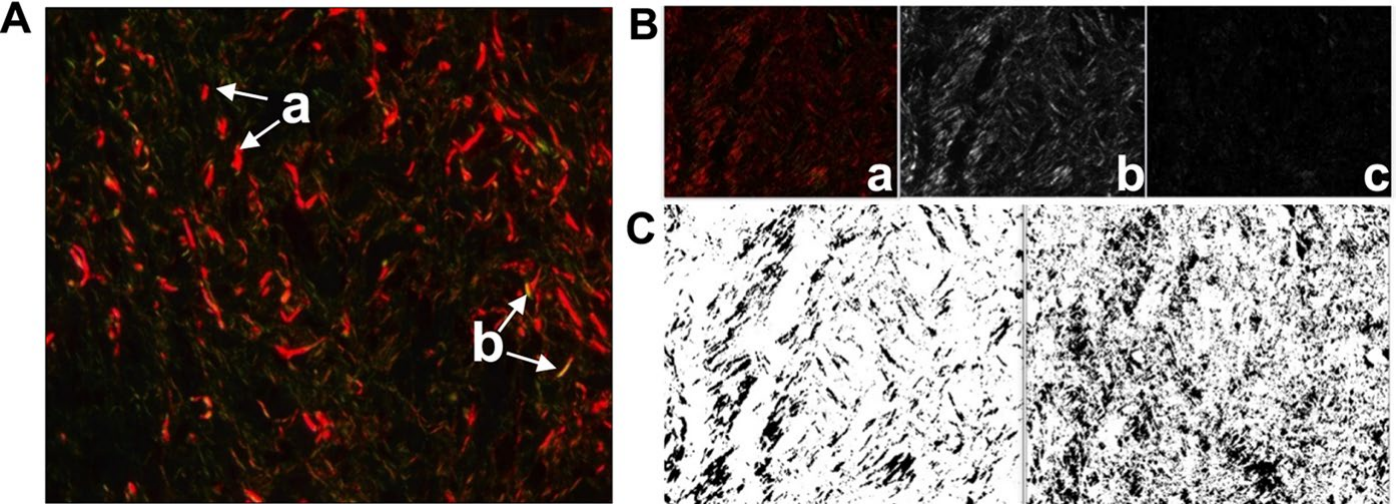
**A** Image of birefringent collagen fibers stained with picrosirius obtained by polarized light microscopy. In the image it is possible to visualize red (**a**) and green (**b**) birefringent collagen fibers, indicated by arrow. Increase by 40×. **B** Representation of the process *Split Channel*: a–Original RGB Image; Split image for the red and green components respectively b and c. **C** Representation of the binarization process. The image becomes black and white. Collagen fibers appear in black and the rest of the image is blank

Regarding type III collagen, DF analysis showed a higher mean for groups treated with homologous and heterologous PRP compared to the control (Fig. 2). No major differences were observed between type I and type III collagens in the treated groups.

Concerning the total area of the collagen (Table 1), without differentiating between type I and type III fibers, and after comparison between treated and control groups, a significant increase was observed in the autologous (*p* = 0.009) and heterologous (*p* = 0.016) PRP groups. In the homologous group, no major differences were observed, and the mean of the collagen area in this group was lower than in the control group. However, the Kruskall-Wallis test showed a difference in the autologous group compared to the homologous group (*p* = 0.016), as well as the heterologous group (*p* = 0.040). There was no correlation between the treated groups and controls in non- parametric analysis.

Although the proliferation of fibroblasts and collagenization are generally physiologically proportional, in this study it was observed that the groups that received autologous and heterologous PRP treatments showed a greater area of collagen, as well as a higher density of type I collagen in DF analysis compared to the controls.

In the autologous group compared to the control group, we observed an increase of 34.76% in the total collagen area (*p* = 0.009), in addition, there was an increase of 0.04 in the median density of type I collagen (*p* = 0.028). In relation of heterologous, it showed an area 12.679 pixels bigger compared to the control group (*p* = 0.016), as the collagen type 1 density with an increase of 0.2 points of median. Even though those parameters increased, collagen type III to entire groups did not show relevant variability; also, there were no difference among the others parameters in homologous group.

When compared to the autologous and heterologous groups, the PRP obtained by the homologous group demonstrated variability on the parameters evaluated, with an increase in collagen type I and III density and a reduction in the total area and fibroblasts numbers. Still, these findings are not relevant for its *p*-value > 0.05.

Optical microscopy analysis on HE stained slides did not show any signs of necrosis or inflammation in all groups.

## Discussion

These findings contradict the results of Marques et al. (2017) and Moreira et al. (2016), which although they used different evaluation methods, did not observe an increase in collagen in dermal tissues treated with heterologous and autologous PRP, respectively.

Regarding the contradictions and variability of the results obtained with PRP, the literature suggests that this variability arises from differences in the preparation processes (Tey et al. 2022), such as centrifugation time and speed (Croisé et al. 2020), which can result in PRP products with distinct biochemical compositions (Simental-Mendía et al. 2023).

The concentration of platelets and leukocytes can vary significantly between preparations, and these differences may influence clinical outcomes (Boffa et al. 2024). Some studies suggest that different types of PRP (rich or poor in leukocytes) may have distinct effects, and the method by which PRP is activated (or not activated) is also a source of heterogeneity in these studies (Wu et al. 2025; Simental-Mendía et al. 2023). This significant heterogeneity in preparation methods (centrifugation protocols, leukocyte concentrations, activation techniques, dosing) and study designs is explicitly pointed out as a major limitation in systematic reviews (Fang et al. 2020; Wu et al. 2025; Xu et al. 2020; Lim et al. 2025). Therefore, this lack of standardization hinders the comparison between results from different studies and may be the underlying reason for the contradictions we observe about previous studies.

Another study, Ferraciolli et al. (2018) also observed an increase in collagen fibers within the autologous group. These studies evaluated collagen in a general way, without differentiating between type I and type III. When evaluating collagen ‘in general’ or using qualitative/subjective methods, some studies (Marques et al. 2017; Moreira 2016; Ferraciolli et al. 2018) may have missed important nuances in the organization and differential deposition of Type I and Type III collagens that the Fractal Dimension analysis was able to capture (Lima et al. 2023; Bielajew et al. 2020). Therefore, the ability of DF to assess the complexity and irregularity of collagen fibers may have been crucial in identifying the differences in the organization of Type I collagen observed in the autologous group.

In our study, the homologous group did not show a significant increase in the total collagen area or the density of Type I, and the fibroblast count also did not increase significantly. This lower response observed with homologous PRP, compared to autologous and heterologous, may be attributed to a combination of factors such as variability in the composition of PRP depending on the origin (individual/species) (Gupta et al. 2021; Masuki et al. 2016; Meira et al. 2020) and probably an immune response of the recipient rabbit to the components of PRP from another individual (homologous), which is a documented concern for allogenic PRP (Gupta et al. 2021). Although heterologous PRP also introduces foreign material (Gupta et al. 2021), the nature and impact of the immune response to PRP from another species (dog to rabbit) may be different or less harmful in the context of wound healing in this specific experimental model (Wang et al. 2025), perhaps offset by a particularly favorable growth factor profile of canine PRP.

Regarding the organization of the fibers, DF analysis also showed that the groups that received PRP treatment had more organized and homogeneous collagen fibers, which is consistent with studies in literature (O'Dowd 2022; Ferraciolli et al. 2018). Another interesting finding was described (Vocca et al. 2023) regarding heterologous PRP, which presents a peripheral mechanism of action, showing a potent antinociceptive activity, which supports the use of biomaterial type in dermal lesions.

Optical microscopy analysis on HE stained slides did not show any signs of necrosis or inflammation in the tissues analyzed this is consistent with previous studies (Zheng et al. 2022; Palacio et al. 2016).

In view of this, there are two possible explanations: either a delay occurred in the healing of the control groups because, in other analyses in this study, it was confirmed that the treated groups had a greater increase in collagen; or the controls may form an exuberant and hypertrophic scar over time, as the increase in fibroblasts indicates they may still be proliferating and synthesizing collagen (Boraldi et al. 2024). The results observed in the groups treated with PRP (autologous and heterologous) showed greater total collagenization and higher density of Type I collagen, indicating a potentially more regulated healing process with a lower risk of pathological scar formation. PRP, being a rich source of growth factors and cytokines, has the potential to modulate the regenerative microenvironment, promote tissue anabolism, and potentially reduce inflammation. This modulation can guide the healing process towards a more efficient maturation, with the appropriate transition and robust deposition of Type I collagen, which is the main component of mature and organized scar tissue, resulting in a scar with a lower likelihood of developing excessive fibrosis and the structural disorganization typical of hypertrophic scars (Pu et al. 2023).

In view of such results, the assumption can be made that autologous and heterologous sources have a greater capacity to mediate and accelerate the healing process compared to homologous treatments. However, further testing is needed to validate this hypothesis.

## Conclusion

DF analysis proved to be an efficient method for characterizing collagen in experimentally induced dermal wounds. The methods used in this study to evaluate the production of collagen demonstrated that the use of PRP gel from different sources for the treatment of dermal wounds is efficient, but the autologous gel demonstrated better collagenation when compared to heterologous and homologous.

## Supplementary Information

Below is the link to the electronic supplementary material.

Supplementary file1 (DOCX 32 kb)

## Data Availability

No datasets were generated or analysed during the current study.
