# Supplementary figures and images for: Quantification and characterization of collagen in experimentally induced wounds treated with different types of platelet-rich plasma gel
Source: Cell Tissue Bank. 2026 Mar 4;27(2):15. doi: 10.1007/s10561-026-10215-5 (PMC12960424; doi:10.1007/s10561-026-10215-5)

**Supplementary data:**

**
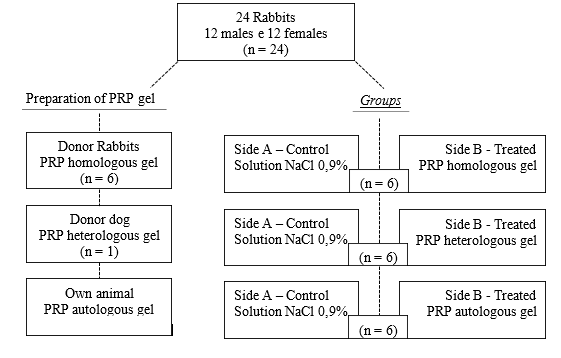
**

Supplement: Supplementary file 1 — Supplementary file1 (DOCX 32 kb) [file 10561_2026_10215_MOESM1_ESM.docx]
